# Protective Effects of PPAR*γ* on Renal Ischemia-Reperfusion Injury by Regulating miR-21

**DOI:** 10.1155/2022/7142314

**Published:** 2022-08-30

**Authors:** Ruizhen Huang, Cong Zou, Chiyu Zhang, Xing Wang, Xin Zou, Zhengjie Xiang, Zewei Wang, Bin Gui, Tao Lin, Honglin Hu

**Affiliations:** ^1^Department of Urology, The Second Affiliated Hospital of Nanchang University, Nanchang, China; ^2^Department of Endocrinology, The Fourth Affiliated Hospital of Nanchang University, Nanchang, China

## Abstract

Renal ischemia-reperfusion injury (RIRI) is a common pathological process that causes kidney injury. Previous studies have indicated that both peroxisome proliferator-activated receptor *γ* (PPAR*γ*) and microRNA-21 (miR-21) exert protective effects against RIRI. However, their relationship is not well understood. In the present study, we investigated the role of the PPAR*γ*/miR-21/programmed cell death 4 (PDCD4) axis in IRI, both *in vivo* and *in vitro*. *In vitro* cell hypoxia/reoxygenation (H/R) and *in vivo* RIRI models were established, and cell viability, cell apoptosis, and key molecule expression profiles were analyzed. Our results showed that both PPAR*γ* and miR-21 had protective effects against RIRI to varying degrees, and there was an interaction between PPAR*γ* and miR-21. PPAR*γ* could promote the expression of miR-21 and partially protect against RIRI by reducing the level of the miR-21 target protein (PDCD4). Our findings underscore the potential utility of future clinical investigations of PPAR*γ* activation and targeting of the underlying miR-21/PDCD4/caspase-3 pathway, which may participate in the pathogenesis of human IRI.

## 1. Introduction

Renal ischemia-reperfusion injury (RIRI) is a relatively common pathological process that causes kidney injury and severe complications such as acute renal failure. Complicated molecular mechanisms, including the accumulation of reactive oxygen species (ROS), apoptosis, and inflammatory cell infiltration, are reported to be pivotal in the process of RIRI [1, 2]. However, elaborating the exact mechanisms of RIRI is difficult, which makes RIRI a challenge during clinical therapy.

Activation of peroxisome proliferator-activated receptor *γ* (PPAR*γ*) is important in protecting organs from IRI [3]. As kidneys have an abundant blood supply, they are sensitive to ischemia, and even transient ischemia caused by various reasons may induce severe renal reperfusion injury. RIRI is associated with high morbidity and mortality. We previously demonstrated that the PPAR*γ* synthetic ligand pioglitazone exerts its renoprotective functions by inhibiting apoptosis, alleviating oxidative stress, and enhancing autophagy in *in vitro* and *in vivo* models [4–6]. Moreover, it seems that RIRI-induced inflammatory and oxidative responses are potentially ameliorated by administering pioglitazone, with inhibition of the NF-*κ*B signaling pathway, and alleviated renal insults in rats [7]. The crucial roles of modulating NF-*κ*B signaling pathway-mediated inflammation and oxidative stress in IRI have also been demonstrated by other researchers [8, 9], which may provide a theoretical basis for the exploration of novel targets and pathways in RIRI therapy. In this study, we further investigated the underlying molecular mechanisms of the nuclear transcription factor PPAR*γ* in RIRI.

As an important member of the microRNA family, microRNA-21 (miR-21) has been studied as a tumor-related gene in previous studies. Recent studies on miRNA-21 have shown that it is also closely associated with the pathological process of IRI. Since Godwin et al. identified the miRNA signature of RIRI [10], substantial efforts have been made to understand the relationship between miRNAs and organ IRI amelioration. The protective effects of miR-21 in RIRI have been demonstrated in our previous studies, and the levels of blood urea nitrogen (BUN) and creatinine in the miR-21 overexpression group were lower than those in the corresponding control group [11]. We explored the effects of miR-21 and PPAR*γ* as well as the crosstalk between miR-21 and PPAR*γ*.

## 2. Materials and Methods

### 2.1. Cell Culture

The NRK-52E cell line was provided by *Procell Life Science & Technology Co., Ltd.* The cells were cultured in Dulbecco's modified Eagle's medium (DMEM) containing 1% penicillin-streptomycin and 10% fetal bovine serum (FBS; Gibco, Grand Island, NY, USA) in T-25 culture flasks at 37°C under 5% CO_2_.

### 2.2. Cell Transfection

PPAR*γ* overexpression (OE) plasmid or shRNA (Hanbio, Shanghai, China) was transfected to regulate PPAR*γ* expression. miR-21 mimic and inhibitor (Hanbio, Shanghai, China) were transfected to regulate miR-21 expression. For cell transfection, Lipo3000 transfection reagent (Invitrogen, Carlsbad, CA, USA) was used, according to the manufacturer's instructions.

### 2.3. H/R Protocol

The cells were cultured to the logarithmic phase, and the H/R protocol was performed as follows. The hypoxic condition was realized in a constant temperature three-gas cell incubator, and the incubator was subjected to 95% N_2_ and 5% CO_2_ for 4 h. For reoxygenation, the culture medium of hypoxic cells was replaced, and the cells were returned to the normoxic culture chamber at 37°C under 5% CO_2_ conditions for 12 h.

### 2.4. Cell Counting Kit-8 (CCK-8) Assay

To investigate cell viability, a CCK-8 (US Everbright Inc., China) assay was performed. Cells were seeded at a density of 8000 cells/well into 96-well plates before the assay was performed. The reagent (10 *μ*L) was added after incubation at 37°C for 2 h, and a microplate reader was added; the absorbance was measured at a wavelength of 450 nm (A450).

### 2.5. Determination of Tumor Necrosis Factor-*α* (TNF-*α*) and Lactate Dehydrogenase (LDH) Levels

The cell culture medium was collected for each group. LDH commercial kit reagents (Solarbio, Beijing, China) were used for determination. All experimental procedures were performed in accordance with the manufacturer's instructions. TNF-*α* levels were detected using an enzyme-linked immunosorbent assay (ELISA) kit (Boster, Wuhan, China) according to the manufacturer's instructions.

### 2.6. Flow Cytometric Analysis

Cells were washed twice with PBS, harvested with trypsin solution without EDTA, collected, and centrifuged at 1,000 rpm for 5 min. The apoptosis rate was detected for the resulting cells. All procedures were based on FITC-Annexin V and PI Apoptosis Kit (US Everbright Inc., China). After mixing with 300 *μ*L of prechilled 1x binding buffer for each tube, further detection was realized using a flow cytometer.

### 2.7. Dual-Luciferase Reporter Assay

To confirm the relationship between miR-21 and the target genes, the synthesized wild-type (wt) and mutant (mu) luciferase reporter plasmids (Hanbio, Shanghai, China) were used for luciferase reporter assays. The prepared NRK-52E cells were seeded in a six-well plate, and after the cells reached 80% confluence, cotransfections of wt-PDCD4 or mu-PDCD4 plasmids and miR-21 mimics or miR-21 negative controls were performed using Lipofectamine 3000 (Thermo Fisher, USA). Six hours later, the culture medium was replaced, and 48 h after transfection, the cells were collected for detection. Dual-luciferase activity was detected using a commercial Dual-Luciferase Reporter Assay Kit (TransGen Biotech, Beijing, China) according to the manufacturer's instructions.

### 2.8. Western Blotting

Total protein of NRK-52E cells for each group was extracted using a RIPA lysis solution, and a bicinchoninic acid (BCA) protein determination assay (Solarbio, Beijing, China) was used to examine the protein concentrations and was kept consistent. Proteins were separated by electrophoresis on a 10% sodium dodecyl sulfate- (SDS-) polyacrylamide gel. The proteins were transferred to polyvinylidene fluoride (PVDF) membranes, and 5% skimmed milk was used to seal the PVDF membrane at room temperature for 1 h. Then, the anti-PPAR*γ* (Proteintech, Wuhan, China, 1 : 2000), anti-PDCD4 (Proteintech, Wuhan, China, 1 : 1000), anti-caspase-3 (Proteintech, Wuhan, China, 1 : 200), and anti-GAPDH (Proteintech, Wuhan, China, 1 : 5000) primary antibodies were incubated overnight at a temperature of 4°C. The next day, after incubation with HRP-conjugated secondary antibodies, an ECL (US Everbright, China) solution was used for further analysis.

### 2.9. Real-Time PCR Assay

Total RNA was extracted using the TransZol Up reagent (TransGen Biotech, Beijing, China). The concentrations and purity of the samples were determined using an ultraviolet spectrophotometer. Reverse transcription was performed to synthesize cDNA using the EasyScript® One-Step gDNA Removal and cDNA Synthesis SuperMix reagent Kit (TransGen Biotech, Beijing, China) according to the manufacturer's instructions, and the SYBR Premix Ex Taq kit (Takara, Dalian, China) was then used for RT-PCR. The following sequences were used: GAPDH (sense: 5′-GAAAGACAACCAGGCCATCAG-3′, antisense: 5′-TCATGAATGCATCCTTTTTTGC-3′), U6 (sense: 5′-CTCGCTTCGGCAGCACA-3′, antisense: 5′-AACGCTTCACGAATTTGCGT-3′), PPAR*γ* (sense: 5′-GGACGCTGAAGAAGAGACCTG-3′, antisense: 5′-CCGGGTCCTGTCTGAGTATG-3′), PDCD4 (sense: 5′-TTGAGCACGGAGATACGAAC-3′, antisense: 5′-GTCCCGCAAAGGTCAGAAAG-3′), and miR-21 (sense: 5′-GCCCGCTAGCTTATCAGACTGATG-3′, antisense: 5′-GTGCAGGGTCCGAGGT-3′).

### 2.10. Animal Procedures

Pathogen-free male C57BL/6 mice (8–10 weeks old, weighing 18–20 g) were obtained from the Laboratory Animal Center of Nanchang University (Nanchang, Jiangxi, China). This study was assessed and approved by the Ethics Committee for Animal Experiments of the Second Affiliated Hospital of Nanchang University. For renal transfection, the designed overexpression PPAR*γ* recombinant adeno-associated virus (AAV9) (Hanbio, Shanghai, China) was injected directly into the kidneys. Preoperative fasting was performed for 8–12 h before surgery, and the rats were anesthetized intraperitoneally by administering pentobarbital sodium (50 mg/kg). When fully anesthetized, the left kidney was exposed via a 1.5–2 cm incision along the median abdominal line, and vehicles were injected into three to four sites with recombinant AAV [12]. Four weeks after the injection, antagomiR-21 and premiR-21 (RiboBio, Guangzhou, China) were used for miR-21 regulation. AntagomiR-21 and premiR-21 were injected via tail veins into the mice for 3 consecutive days. The mice were sacrificed for kidney isolation, quickly frozen in liquid nitrogen, and stored at –80°C for further analysis. The PPAR*γ*-AAV9 transfection efficiency and miR-21 expression levels were measured.

### 2.11. Mouse Model of RIRI

Mice were abstained for 8–12 h before the construction of the RIRI model. The mice were anesthetized by intraperitoneal administration of pentobarbital sodium (50 mg/kg). Following a 1.5–2 cm incision along the median abdominal line, the renal pedicles were bluntly dissected, and RIRI was induced by bilateral clamping of the renal arteries. Once the color of the kidneys turned into a paler shade, occlusion was verified. After the kidneys were subjected to pedicle occlusion for 45 min, the microvascular clamps were removed from the pedicles, and the reperfusion stage was immediately implemented. Mice were sacrificed for further analysis 24 h after reperfusion.

### 2.12. Assessment of Renal Function

Blood samples were collected from the orbital vein of the mice into centrifuge tubes 24 h after reperfusion. Serum was obtained by centrifuging the blood samples at 3,000 rpm for 10 min. Serum creatinine (Scr) and blood urea nitrogen (BUN) levels were examined according to the manufacturer's instructions to assess renal function, and commercial detection kits were obtained from the Nanjing Jiancheng Bioengineering Institute (Nanjing, China).

### 2.13. Histopathologic Evaluation of Kidney

The kidneys of euthanized mice in different groups were collected 24 h after reperfusion. For histopathological examination, the kidneys were dissected coronally and fixed using a paraformaldehyde tissue fixator, embedded in paraffin, and sliced into 4 mm sections for hematoxylin and eosin (H&E) staining. Morphological changes in renal tissue were observed using an optical microscope. The degree of renal tubular necrosis was assessed according to the method described by Zou et al. [7]. A maximum score of 4 indicates the highest degree of necrosis, 0 = normal kidney; 1 = minimal necrosis, <5% necrotic renal tubular involvement; 2 = mild necrosis, 5%–25% involvement; 3 = moderate necrosis, 25%–75% involvement; and 4 = severe, >75% involvement.

### 2.14. Immunohistochemical (IHC) Analysis

An IHC assay was used to examine PPAR*γ* and PDCD4 expression in the kidneys. The sacrificed kidney tissues were embedded in paraffin and cut into 4 *μ*m thick sections. The samples were deparaffinised with xylene and rehydrated with pure ethanol. BSA (3%) was added to the circle to evenly cover the tissue sections, which were then sealed for 60 min at room temperature. The sealing solution was gently removed, and the PPAR*γ* (Cell Signalling Technology; USA) and PDCD4 (Cell Signalling Technology; USA) primary antibodies were used at a dilution of 1 : 200 for incubation overnight at 4°C. The sections were placed in PBS and washed by shaking on a decolorizing shaker thrice for 5 min each. After the sections were shaken slightly and dried, the tissues were covered with HRP-labeled secondary antibodies. After incubation with DAB and counterstaining with hematoxylin, the images were collected using light microscopy.

### 2.15. Immunofluorescence Analysis

Kidney tissues were embedded in paraffin and cut into 4 *μ*m slides. The samples were deparaffinised with xylene and rehydrated with pure ethanol. Blocking was performed with 5% bovine serum albumin for 2 h at room temperature. The sealing solution was gently removed; slides were incubated with the PPAR*γ* primary antibodies (1 : 200; Cell Signalling Technology; USA) and PDCD4 (1 : 600; Cell Signalling Technology; USA) primary antibody overnight at 4°C. The slides were washed three times with PBS, and then, the objective tissue was covered with a secondary antibody and incubated at room temperature for 60 min in the dark. After incubation with the DAPI solution at room temperature for 20 min in the dark, images were acquired using a fluorescence microscope.

### 2.16. TUNEL Assay

The terminal deoxynucleotidyl transferase-mediated dUTP nick end labeling assay (TUNEL) was used for examining cell apoptosis. The sacrificed kidney tissues were embedded in paraffin and cut into 4 *μ*m thick sections. The samples were deparaffinised with xylene and rehydrated with pure ethanol. A 0.5% Triton-X-100 working solution was used for permeabilization, incubated at room temperature for 20 min, and washed thrice with PBS. The TUNEL reaction was performed according to the instructions of the TUNEL Detection Kit (Servicebio, Wuhan, China). After incubation with the DAPI solution at room temperature for 10 min, images were collected using a fluorescence microscope.

### 2.17. Statistical Analysis

All data were statistically analyzed using the GraphPad Prism software (version 8.0). Quantitative data were expressed as mean (*x*) ± S.E. Differences between two groups were compared using the *t*-test, and multiple groups were compared using one-way ANOVA along with Tukey's post hoc multiple-comparisons test. *p* < 0.05 was considered statistically significant.

## 3. Results

### 3.1. PPAR*γ* and miR-21 Improved Cell Viability after the H/R Procedure

**Figure 1:**
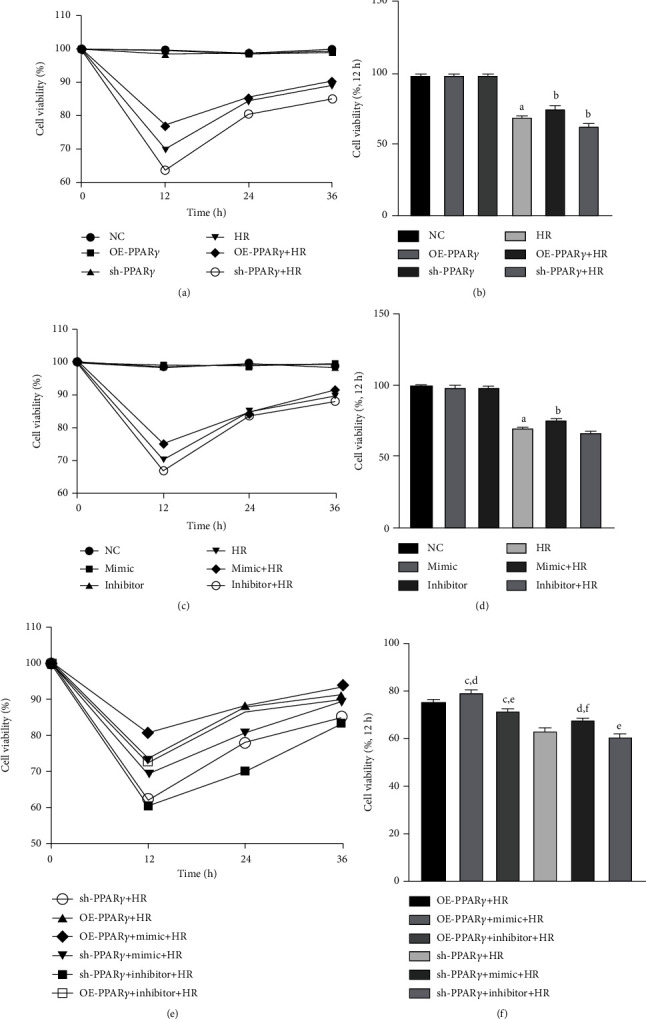
Cell viability of each group, detected after 12 h reoxygenation: (a) cell viability of PPAR*γ* regulation groups (*n* = 4); (b) column chart of PPAR*γ* regulation groups; (c) cell viability of miR-21 regulation groups; (d) column chart of miR-21 regulation groups; (e) cell viability of the cotransfection groups; (f) column chart of the cotransfection groups (A represents values compared to the NC group, *p* < 0.05; B represents values compared to the HR group, *p* < 0.05; C represents values compared to the OE-PPAR*γ*+HR group, *p* < 0.05; D represents values compared to the mimic+HR group, *p* < 0.05; E represents values compared to the inhibitor+HR group, *p* < 0.05; F represents values compared to the sh-PPAR*γ*+HR group, *p* < 0.05).

A CCK8 assay was performed to evaluate the viability of each group. As shown in Figure 1, cells subjected to H/R showed significantly decreased cell viability compared with the negative control (NC) group. However, cell viability in cells subjected to H/R was significantly improved when PPAR*γ* and miR-21 were upregulated as compared to that in the H/R alone group. Interestingly, when both PPAR*γ* and miR-21 were upregulated simultaneously in the NRK-52E cells, cell viability was significantly improved compared to that in the H/R group. miR-21 may have little effect on cytoprotection under conditions of low PPAR*γ* expression.

### 3.2. Regulation of PPAR*γ* and miR-21 Influences LDH and TNF-*α* Levels after H/R

**Figure 2:**
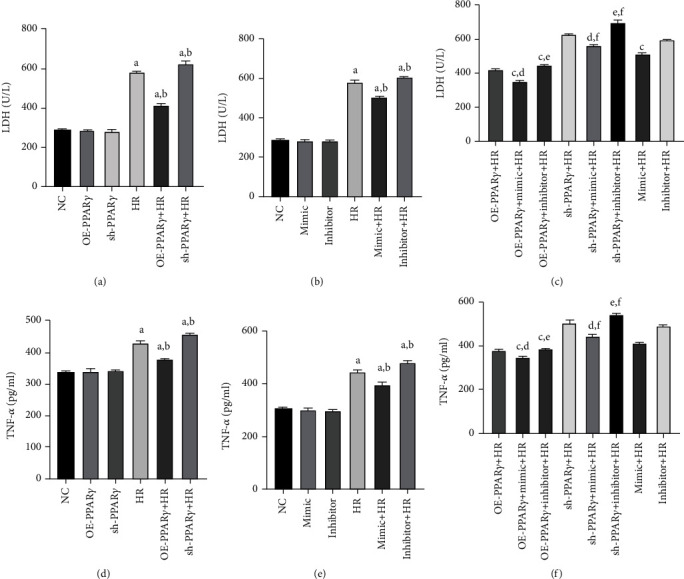
LDH and TNF-*α* levels: (a) LDH level of PPAR*γ* regulation groups (*n* = 4); (b) LDH level of miR-21 regulation groups; (c) LDH level of the cotransfection groups; (d) TNF-*α* level of PPAR*γ* regulation groups; (e) TNF-*α* level of miR-21 regulation groups; (f) TNF-*α* level of the cotransfection groups. LDH and TNF-*α* levels were detected after 12 h reoxygenation (A represents values compared to the NC group, *p* < 0.05; B represents values compared to the HR group, *p* < 0.05; C represents values compared to the OE-PPAR*γ*+HR group, *p* < 0.05; D represents values compared to the mimic+HR group, *p* < 0.05; E represents values compared to the inhibitor+HR group, *p* < 0.05; F represents values compared to the sh-PPAR*γ*+HR group, *p* < 0.05).

The LDH and TNF-*α* levels were measured to evaluate the cytoprotective effects of PPAR*γ* and miR-21 against H/R injury. In cells subjected to H/R injury, LDH and TNF-*α* are released from intracellular organelles. Therefore, LDH and TNF-*α* levels were examined to assess the degree of H/R injury. Levels of LDH and TNF-*α* increased dramatically in the H/R group, and PPAR*γ* and miR-21 had a cytoprotective effect. Overexpression of PPAR*γ* significantly decreased cell injury, whereas interfering with PPAR*γ* expression led to more severe cell injury than in the H/R group (Figures 2(a) and 2(d)). miR-21 showed a similar cytoprotective effect (Figures 2(b) and 2(e)). Furthermore, cotransfection with PPAR*γ* and miR-21 resulted in lower LDH and TNF-*α* levels when subjected to H/R injury. However, the protective effect was weakened in the cotransfection groups when either PPAR*γ* or miR-21 was downregulated (Figures 2(c) and 2(f)).

### 3.3. PPAR*γ* and miR-21 Could Mitigate Cell Apoptosis after the H/R Procedure

**Figure 3:**
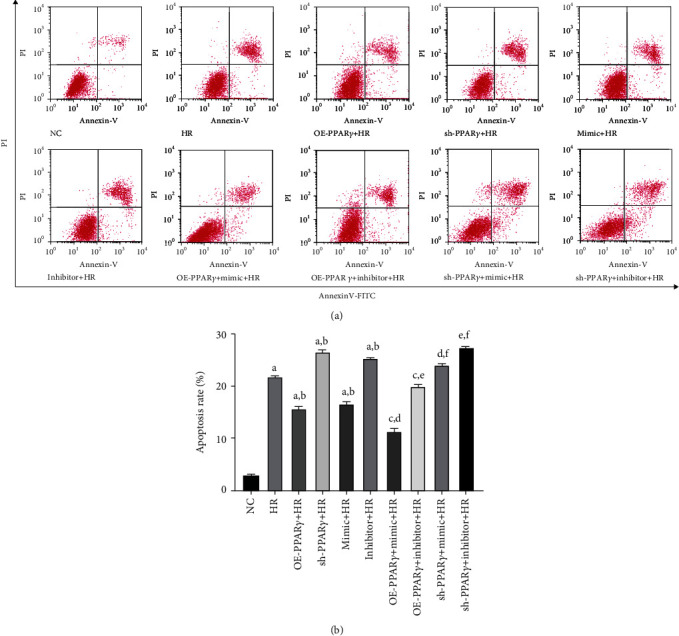
Flow cytometric analysis was used to examine cell apoptosis (*n* = 4): (a) representative images of flow cytometric analysis; (b) quantitative analysis of apoptosis rate (A represents values compared to the NC group, *p* < 0.05; B represents values compared to the HR group, *p* < 0.05; C represents values compared to the OE-PPAR*γ*+HR group, *p* < 0.05; D represents values compared to the mimic+HR group, *p* < 0.05; E represents values compared to the inhibitor+HR group, *p* < 0.05; F represents values compared to the sh-PPAR*γ*+HR group, *p* < 0.05).

To further determine the cytoprotective effects of PPAR*γ* and miR-21, flow cytometric analysis was performed to examine the NRK-52E cell apoptosis levels in each group (Figure 3). Our study indicated that the overexpression of PPAR*γ* and miR-21 reduced the proportion of apoptotic cells during H/R. When PPAR*γ* and miR-21 were upregulated simultaneously, apoptosis in NRK-52E cells during H/R was ameliorated more than merely upregulating PPAR*γ* or miR-21. However, silencing of PPAR*γ* partially reversed this protection, even upregulating miR-21 expression, and cell apoptosis was worse than that in the mimic+HR group. These results suggest that PPAR*γ* and miR-21 could attenuate cell apoptosis, indicating the pivotal role of PPAR*γ* in cytoprotection.

### 3.4. PPAR*γ* Could Upregulate the miR-21 Levels

**Figure 4:**
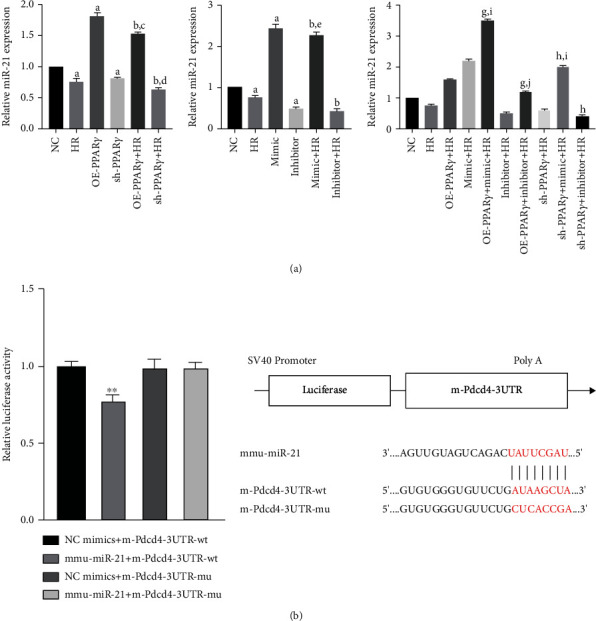
The miR-21 expression and dual-luciferase reporter analysis (*n* = 4): (a) the qPCR analysis of miR-21 expression in each group; (b) dual-luciferase reporter analysis between miR-21 and PDCD4 (A represents values compared to the NC group, *p* < 0.05; B represents values compared to the HR group, *p* < 0.05; C represents values compared to the OE-PPAR*γ* group, *p* < 0.05; D represents values compared to the sh-PPAR*γ* group, *p* < 0.05; E represents values compared to the mimic group, *p* < 0.05; F represents values compared to the inhibitor group, *p* < 0.05; G represents values compared to the OE-PPAR*γ*+HR group, *p* < 0.05; H represents values compared to the sh-PPAR*γ*+HR group, *p* < 0.05; I represents values compared to the mimic+HR group, *p* < 0.05; J represents values compared to the inhibitor+HR group, *p* < 0.05; ∗∗ represents values compared to the NC mimics+m-Pdcd4-3′-UTR-wt group, *p* < 0.01).

As the transcription factor PPAR*γ* is involved in different stages of miRNA biogenesis, its protective effects during H/R injury may be due to the underlying cross-talk between PPAR*γ* and miR-21. To identify the mechanism of PPAR*γ* and miR-21 regulation during H/R, we examined gene expression using qPCR (Figure 4(a)). In the H/R group, both PPAR*γ* and miR-21 expression decreased, and the application of miR-21 mimic and inhibitor effectively regulated miR-21 expression, indicating that transfection was efficient. Furthermore, the miR-21 level in the PPAR*γ* overexpression group was higher than that in the NC group, and the miR-21 level was decreased by silencing PPAR*γ* expression, whereas it seems that activation of miR-21 was unable to affect PPAR*γ* mRNA expression. However, no significant difference was observed between the miR-21 mimic and inhibitor groups. These results demonstrate that the transcription factor PPAR*γ* could upregulate miR-21 levels. In addition, the cytoprotection of PPAR*γ* may be partially mediated by the upregulation of miR-21 levels.

To examine the molecular mechanism of PPAR*γ*-mediated protection of cells against H/R injury, a dual-luciferase reporter assay was performed. It was observed that the miR-21 downregulated promoter activity of PDCD4 by directly targeting 3′-UTR. In other words, the cytoprotection effect of PPAR*γ* on cell H/R injury may partially regulate the miR-21 expression and thus downregulated its target PDCD4 (Figure 4(b)).

### 3.5. PPAR*γ* Alleviates H/R Injury by Downregulating Caspase-3 and PDCD4 Levels

**Figure 5:**
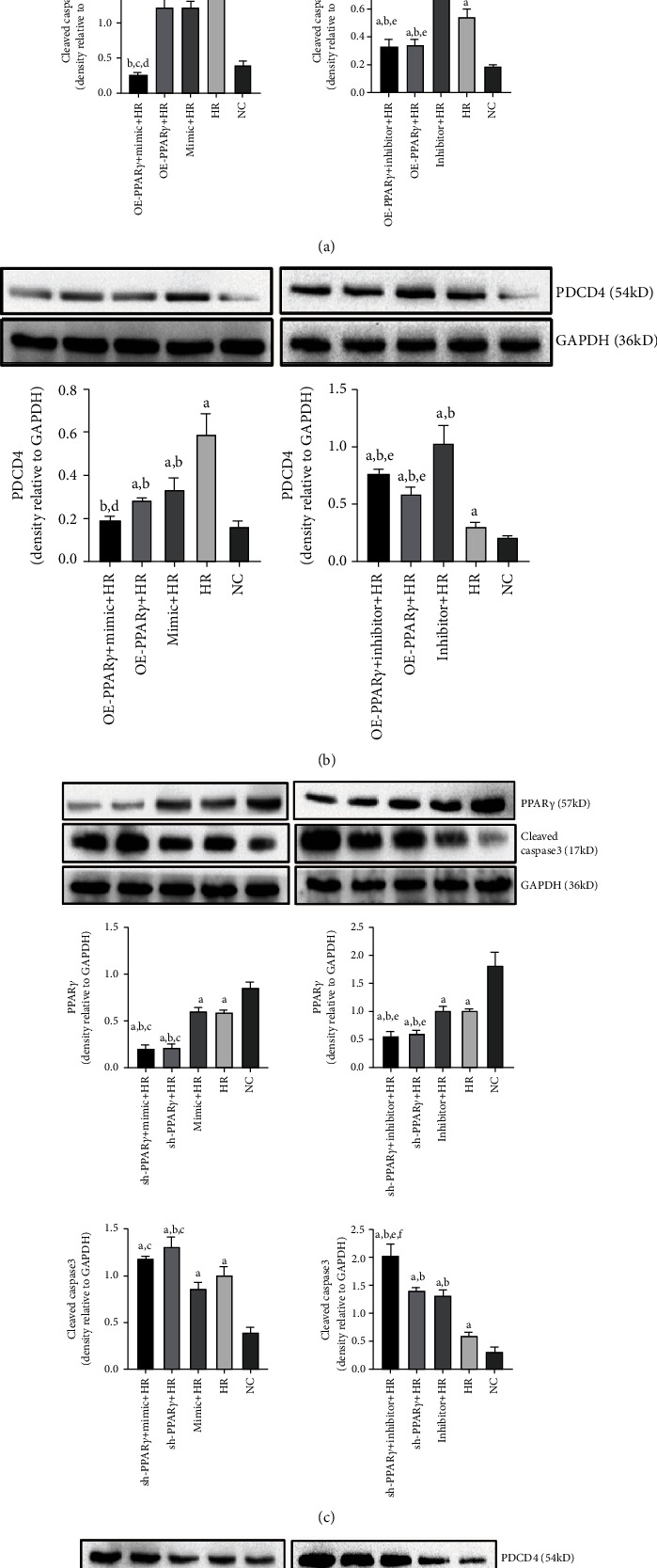
Expression of PPAR*γ*, PDCD4, and cleaved caspase-3 proteins: (a) PPAR*γ* and cleaved caspase-3 protein expressions in PPAR*γ* overregulated and simultaneously miR-21 cotransfection groups; (b) PDCD4 protein expressions in PPAR*γ* overregulated and simultaneously miR-21 cotransfection groups; (c) PPAR*γ* and cleaved caspase-3 protein expressions in PPAR*γ* downregulated and simultaneously miR-21 cotransfection groups; (d) PDCD4 protein expressions in PPAR*γ* downregulated and simultaneously miR-21 cotransfection groups (A represents comparison with NC group, *p* < 0.05; B represents comparison with HR group, *p* < 0.05; C represents comparison with mimic+HR group, *p* < 0.05; D represents comparison with OE-PPAR*γ*+HR group, *p* < 0.05; E represents comparison with inhibitor+HR group, *p* < 0.05).

In the caspase-dependent apoptosis pathway, caspase-3 is the key molecule that stimulates cell apoptosis. PDCD4 is also regarded as a preapoptotic gene. To ascertain the role of PPAR*γ* and miR-21 in H/R injury, we examined the expressions of PPAR*γ*, caspase-3, and PDCD4 using western blotting. We found that H/R induced downregulation of PPAR*γ* proteins, and no significant difference was noted between the miR-21 mimic and inhibitor groups (data not shown). The expression of cleaved caspase-3 and PDCD4 was significantly higher in cells subjected to H/R injury than in the NC group. The overexpression of PPAR*γ* partially reversed their expression, and cleaved caspase-3 and PDCD4 were increased when PPAR*γ* expression was knocked down (Supplementary Figure 1). Additionally, miR-21 expression is related to the expression of cleaved caspase-3 and PDCD4. Upregulated miR-21 expression in the mimic group was accompanied by a decrease in PDCD4 expression, which was blocked by the miR-21 inhibitor (Supplementary Figure 2). In the mimic+HR group, the expression of cleaved caspase-3 and PDCD4 was significantly reduced compared with that in the HR group. When we cotransfected the overexpression PPAR*γ* plasmid and miR-21 mimic, H/R-induced cleaved caspase-3 and PDCD4 expression was significantly ameliorated compared to the OE-PPAR*γ*+HR or miR-21 mimic+HR groups (Figure 5).

### 3.6. PPAR*γ* and miR-21 Improved Kidney Damage in Mice Subjected to I/R Injury

**Figure 6:**
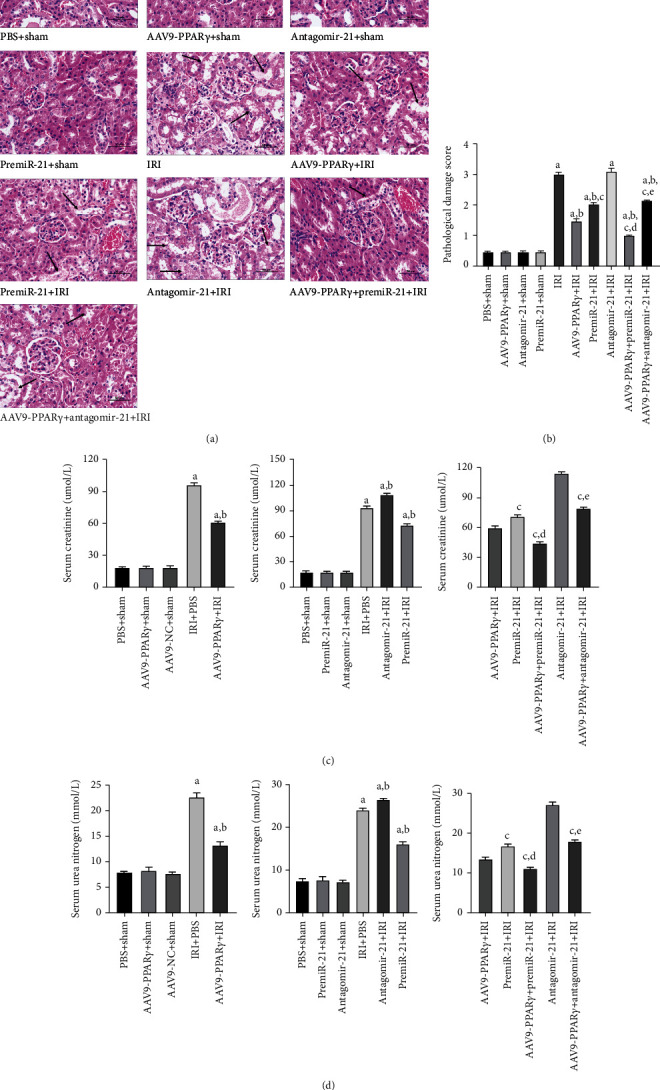
Histological evaluation of the renal tissue 24 h after RIRI and the Scr, BUN levels (*n* = 5). (a) Representative kidney tissues stained with H&E (scale bar 50 *μ*m), arrows indicate tubular dilatation, swelling, congestion, vacuolization, and tubular necrosis in the kidneys. (b) Histologic score of the kidney tissues. (c) Scr levels of each group. (d) BUN levels of each group (A represents values compared to the sham group, *p* < 0.05; B represents values compared to the IRI group, *p* < 0.05; C represents values compared to the AAV9-PPAR*γ*+IRI group, *p* < 0.05; D represents values compared to the premiR-21+IRI group, *p* < 0.05; E represents values compared to the antagomiR-21+IRI group, *p* < 0.05).

A RIRI model was established to investigate the effects of PPAR*γ* and miR-21 on renal tissues. H&E staining was used to evaluate renoprotective effects (Figures 6(a) and 6(b)). IRI resulted in severe histopathological damage in the renal tissue, and serious tubular dilatation, congestion, edema, vacuolization, and tubular necrosis were noted in the IRI group. However, pretreatment with premiR-21 significantly ameliorated this damage. The amelioration of tissue damage was also observed in the PPAR*γ* transfection group, indicating the renoprotective effects of PPAR*γ*.

Scr and BUN levels were used to assess renal function. Collectively, PPAR*γ* and miR-21 could effectively protect the kidney against IRI, and Scr and BUN levels were significantly alleviated in the AAV9-PPAR*γ*+IRI and premiR-21+IRI groups. It is worth noting that despite both PPAR*γ* and miR-21 having renoprotective functions, the AAV9-PPAR*γ*+IRI group had lower Scr and BUN levels than the premiR-21+IRI group, suggesting that PPAR*γ*-mediated miR-21 upregulation was involved in renoprotective effects (Figures 6(c) and 6(d)).

### 3.7. PPAR*γ* and miR-21 Decreased Apoptotic Cell Death in the Kidney Tissue

**Figure 7:**
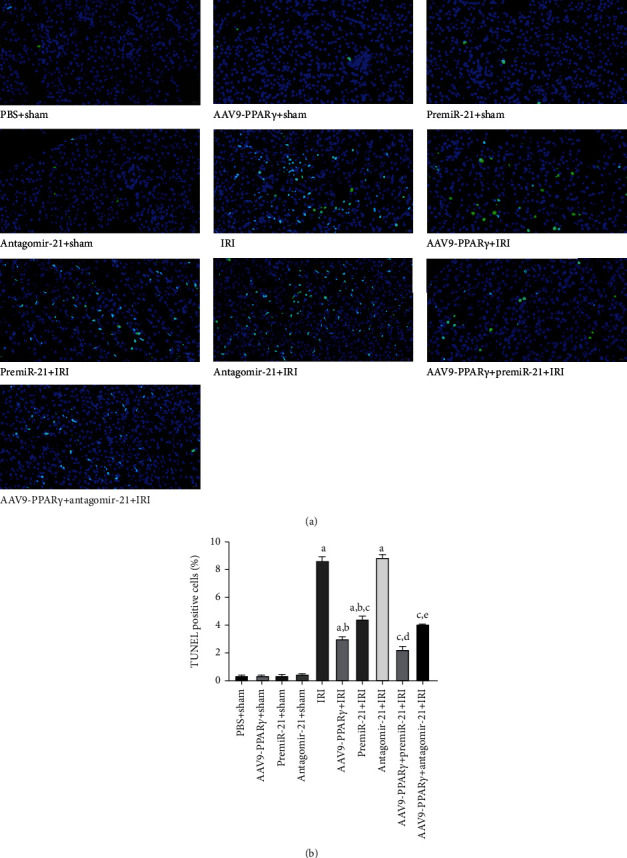
TUNEL assay to evaluate the apoptosis of kidney tissues (*n* = 5): (a) representative photograph of TUNEL assay (scale bar 20 *μ*m); (b) quantitative analysis of TUNEL assay in the kidneys (A represents values compared to the sham group, *p* < 0.05; B represents values compared to the IRI group, *p* < 0.05; C represents values compared to the AAV9-PPAR*γ*+IRI group, *p* < 0.05; D represents values compared to the premiR-21+IRI group, *p* < 0.05; E represents values compared to the antagomiR-21+IRI group, *p* < 0.05).

Groups that underwent IRI had significantly higher apoptosis rates than those in the non-IRI groups. No significant difference in apoptotic cells was observed between the PBS+sham and other sham groups. In comparison with the IRI group, the apoptosis rates in the AAV9-PPAR*γ*+IRI and premiR-21+IRI groups were significantly lower, while the number of apoptotic cells in the antagomiR-21+IRI group was significantly higher. In addition, significant differences were noted in the AAV9-PPAR*γ*+premiR-21+IRI, AAV9-PPAR*γ*+antagomiR-21+IRI, and AAV9-PPAR*γ*+IRI groups. Pretreatment with premiR-21 significantly protected the renal tissues against IRI, and the application of antagomiR-21 resulted in poor renal cell apoptosis. Interestingly, the AAV9-PPAR*γ*+IRI and premiR-21+IRI groups showed that PPAR*γ* activation had a better protective effect than activation of miR-21. These results suggest that PPAR*γ* and miR-21 could decrease apoptotic cell death in kidney tissue and that they play two different roles in renal protection (Figure 7).

### 3.8. Immunohistochemistry and Immunocytochemical Analysis in Kidney

**Figure 8:**
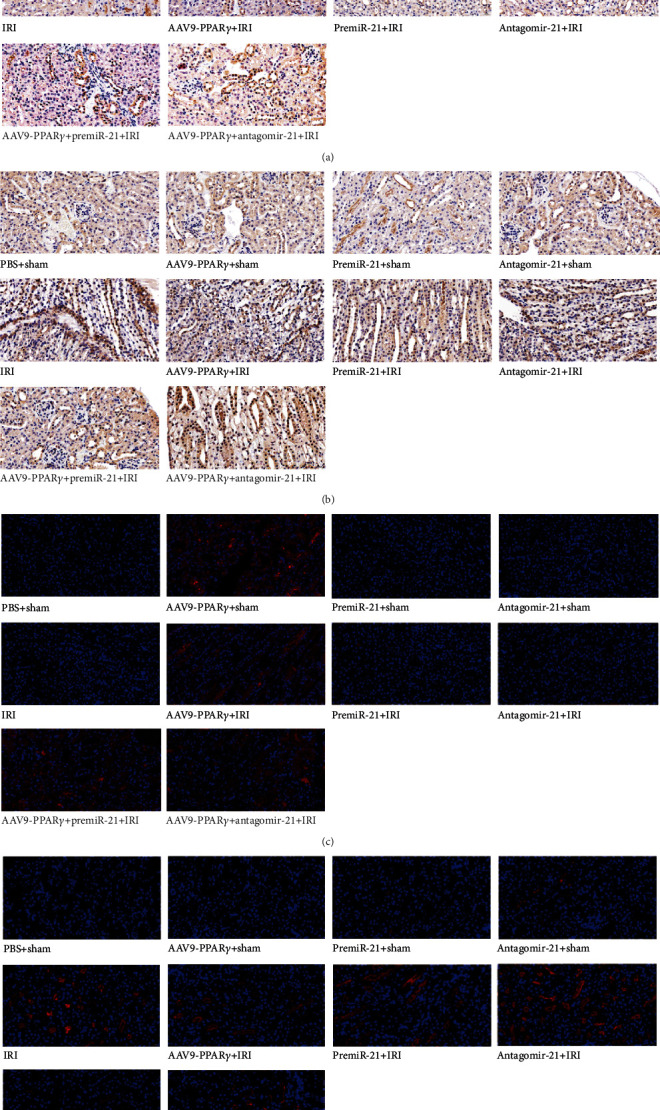
Immunohistochemistry and immunofluorescence analysis in the kidney (*n* = 5): (a) immunohistochemistry analysis of PPAR*γ*; (b) immunohistochemistry analysis of PDCD4 (light-yellow or brown color cells were regarded as positive); (c) immunofluorescence analysis of PPAR*γ*; (d) immunofluorescence analysis of PDCD4 (red was the positive part, all scale bar 20 *μ*m).

PPAR*γ* and PDCD4 expressions were considered the main marker of this study. Comparison of immunohistochemical staining showed that PPAR*γ* expression in the kidney was significantly increased after the PPAR*γ* overexpressed AAV9 was injected directly into the kidney compared to the sham group mice. IRI induced PPAR*γ* expression, whereas miR-21 did not affect PPAR*γ* expression in mouse kidney tissues (Figure 8(a)).

Similar to the western blotting results, PDCD4 expression was significantly increased in mice subjected to RIRI, and PPAR*γ* and miR-21 overexpression was associated with reduced PDCD4 protein levels in mice subjected to RIRI. In contrast, dual overexpression of PPAR*γ* and miR-21 in mice resulted in lower PDCD4 expression than that of the AAV9-PPAR*γ*+IRI and premiR-21+IRI groups, and the PDCD4 was partially reversed by miR-21 blockade (Figure 8(b)).

To investigate the localization of PPAR*γ* and PDCD4 expression, immunofluorescence analysis was performed on the kidney tissues. As shown in the figure, the perinuclear location in kidney tissues was observed during PPAR*γ* expression. Moreover, its expression decreased after being subjected to IRI and was elevated by transfection with AAV9-PPAR*γ*. PDCD4 was expressed in the cellular matrix of kidney tissues. These results suggest that after the ischemic insult in the kidney tissues, PPAR*γ* expression was downregulated and PDCD4 was elevated. Furthermore, no significant difference was noted in PPAR*γ* by premiR-21 and antagomiR-21 pretreatment, whereas at least a portion of PDCD4 was induced by antagomiR-21 and IRI (Figures 8(c) and 8(d)).

## 4. Discussion

The major findings of our study are that in an *in vitro* H/R model, upregulation of PPAR*γ* expression through the plasmid results in the amelioration of H/R injury. Knockdown of PPAR*γ* in NRK-52E cells led to severe H/R injury. Besides, overexpression of miR-21 through miR-21 mimic protects NRK-52E cells against H/R injury, and the protective effect was reversed when miR-21 expression was knocked down by miR-21 inhibitor. Intriguingly, PPAR*γ* appears to be superior to miR-21 in its protective effects. Further research suggests that the superior protective effects of PPAR*γ* may be due to its contribution to the upregulation of the miR-21 levels. The mechanism by which PPAR*γ* and miR-21 exert their effect is likely to be through apoptosis signaling and significant changes in caspase-3. PDCD4 protein expressions reveal that PPAR*γ* ameliorates H/R injury partially via upregulating miR-21 expression and hence decreases miR-21 target PDCD4 expression where caspase-mediated apoptosis signaling is involved. Moreover, PPAR*γ* and miR-21 protected mice against RIRI in an *in vivo* experimental model. These results suggest a potential renoprotective mechanism through which PPAR*γ* mediates the miR-21/PDCD4/caspase-3 axis in RIRI.

PPAR*γ* is a nuclear transcription factor that binds to the peroxisome proliferator responsive element (PPRE) as a heterodimer with retinoid X receptor (RXR). Once activated by its agonists, downstream transcription and expression effects are regulated [13]. The activation of PPAR*γ* leads to the amelioration of renal, hepatic, cerebral, myocardial, and other tissues of IRI [14]. This indicates that PPAR*γ* is a potential therapeutic mediator in IRI. Our previous studies mainly focused on the PPAR*γ* agonist pioglitazone, which was used in type 2 diabetes, to protect against RIRI and the underlying molecular mechanisms. In this study, we directly regulated PPAR*γ* expression via plasmids and found that PPAR*γ* overexpression prevented apoptosis by upregulating miR-21, caspase-3, and PDCD4. The antiapoptosis of PPAR*γ* was also reported by Wu et al. [15] who found that PPAR*γ* overexpression by plasmid had a comparable antiapoptotic effect by upregulating Bcl-2 family proteins, but the antiapoptotic effects were abrogated by PPAR*γ* downregulation when PPAR*γ* siRNA was applied. Another study by Jiang et al. [16] investigated the anti-inflammatory effect of PPAR*γ* on cerebral IRI. In their study, vagus nerve stimulation (VNS) treatment enhanced PPAR*γ* expression, diminished the extent of the ischemic infarct, and alleviated neuronal injury in a rat cerebral IR model. Furthermore, the suppression of inflammatory cytokine secretion during the cerebral IRI process is a result of a PPAR*γ*-mediated mechanism as PPAR*γ* silencing exacerbates ischemic neuronal necrosis. As PPAR*γ* accomplishes these antiapoptotic and anti-inflammatory effects without any exogenous ligands, we speculate that endogenous ligands may be involved in PPAR*γ* activation and thus regulate the expression of noncoding RNA such as miR-21 during the IRI procedure.

miR-21 is upregulated in renal tubular epithelial cells subjected to H/R and in the kidneys following ischemic injury. Knockdown of miR-21 promoted cellular apoptosis and necrosis, whereas miR-21 overexpression downregulated cell death [10]. We previously found that in a RIRI model, IRI induced significant upregulation of miR-21 and exerted its antiapoptotic properties, which may be due to the suppression of PDCD4 and activation of caspase-3/8 fragments [11]. Our present study illustrated similar renoprotective functions of miR-21, and miR-21 was significantly downregulated in our *in vitro* H/R model. Combined with our previous research results, we suggest that miR-21 may be downregulated in the initial stage of IRI, and then, due to the self-defense response, it was upregulated, thereby performing protective functions in the serious disorder stage of cell injury. Researchers have found that in restenosis vascular smooth muscle cells, Bcl-2 is involved in miR-21-mediated cellular effects and miR-21 overexpression is associated with increased Bcl-2 expression [17]. However, research in breast cancer cells has revealed that miR-21 expression is inversely associated with Bcl-2 expression [18], which is fundamentally different from restenosis vascular smooth muscle cells. The controversial role of miR-21 in tissue injury may be correlated with the injury stage, histological differences, and mechanistic distinctiveness.

PDCD4 is a key factor in apoptosis. PDCD4 can activate caspase-3 and initiate apoptosis [19]. The antiapoptotic effect of miR-21 was partly mediated through its target genes PDCD4, and PDCD4 translational suppression by miR-21 has been demonstrated in the pathobiology of cancer cell proliferation [20, 21]. Overexpression of miR-21 reduced PDCD4 promoter activation by directly targeting the 3′-UTR region. Therefore, miR-21 expression was negatively correlated with PDCD4 [22]. These results support the concept that miR-21 functions as a mediator by a mechanism that involves translational repression of PDCD4. Our primary findings demonstrated that upregulation of PPAR*γ* enhanced miR-21 expression, thus inhibiting PDCD4 expression, improving cell viability, and reducing apoptosis in RIRI. PDCD4 may activate caspase-3 and promote cell apoptosis. In contrast, another study suggested that inactivation of miR-21 stimulated PDCD4 and induced pulmonary artery endothelial apoptosis through caspase-3 activation. This phenomenon was abrogated by miR-21 mimic transfection, and the PDCD4/caspase-3 axis was silenced, thus preventing apoptosis [23]. Collectively, our findings demonstrated that the underlying miR-21/PDCD4/caspase-3 axis on activation drives apoptosis both *in vitro* and *in vivo*. From another perspective, the underlying miR-21/PDCD4/caspase-3 pathway may participate in human IRI pathogenesis. Our findings underscore the potential utility of future clinical investigations targeting the miR-21/PDCD4/caspase-3 axis. In a study on pulmonary arterial hypertension, Green et al. [24] found that PPAR*γ* activation can reduce the proliferation of vascular smooth muscle cells and affect disease progression by regulating miR-21 and affecting PDCD4 expression. In conclusion, combined with these results, intrinsic regulation may also exist between PPAR*γ* and miR-21 during RIRI.

As a transcription factor, PPAR*γ* is generally considered to be involved in the regulation of one or more classes of functionally related protein-coding genes, and PPAR*γ* has been identified as a regulator of lipid metabolism and fibrosis [25]. Most miRNAs are transcribed from specific genes with specific promoters located throughout the genome. miRNAs have been confirmed to participate in the pivotal biological processes of posttranscriptional gene regulation [26]. Since transcription factors and miRNAs function as transcription and translation factors, little is known about their relationship. Fortunately, an increasing number of studies have begun to focus on elaborating the molecular mechanisms involved in pathophysiological procedures. In 2015, Dharap et al. used the PPAR*γ* synthetic ligand rosiglitazone in adult rats before miRNA profiling was conducted using microarrays, and 28 miRNAs were found to be altered significantly after treatment with rosiglitazone [27]. A study conducted by Winkler et al. [28] demonstrated that PPAR*γ* directly regulates a class of miRNAs that are functionally associated with antifibrotic properties. In their study, PPAR*γ* was described as a key regulator in antifibrotic miRNA regulation and miRNAs targeting key fibrosis-associated genes. Hence, the formation of an intricate coherent feed-forward loop among PPAR*γ*, miRNAs, and the corresponding target gene plays a significant role in fibrosis regulation. In contrast, PPAR*γ* and miRNAs may have diverse relationships other than the modulatory role of PPAR*γ* in miRNAs. For example, PPAR*γ* was identified as a miR-130b direct target in colorectal cancers [29], and miR-130b was also identified as an independent prognostic biomarker in glioma patients by targeting PPAR*γ* [30]. In addition to miR-130b, miR-27a [31], miR-128 [32], and miR-19b [33] were proved to act as upstream mediators targeting PPAR*γ*. miRNAs may have potential promoter regions in the PPREs. The miR-145 and miR-329 expression levels were significantly modulated by PPAR*γ* expression vector transfection and were reversed by point mutations of PPREs in their promoters. Furthermore, transfection with miR-329 mimic and miR-145 mimic was demonstrated to activate PPAR*γ*, suggesting underlying binding sites for miRNAs in the PPAR*γ* promoter [27]. Mechanistically, miR-21 is regulated by the transcription factor PPAR*γ*. Our study highlights the role of the master regulator of transcription factor PPAR*γ* in miR-21 regulation. However, the regulatory function needs to be comprehensively considered in various pathologies, tissues, and biological procedures before the mechanism whereby PPAR*γ* activation prevents RIRI can be elucidated.

## 5. Conclusion

In this study, we showed that both PPAR*γ* and miR-21 had protective effects on RIRI to varying degrees, overexpression of PPAR*γ* or miR-21 could mitigate the cell damage and cell apoptosis through regulating the apoptotic proteins, and there was an interaction between PPAR*γ* and miR-21. Our findings underscore the potential utility of future clinical investigations, whereby PPAR*γ* activation regulates the underlying miR-21/PDCD4 pathway, which may participate in human IRI pathogenesis.

## Data Availability

The data that support the findings of this study are available from the corresponding author, upon reasonable request.

## Supplementary Materials

Supplementary Materials Supplementary Figure 1: expression of PPAR*γ*, PDCD4, and cleaved caspase-3 proteins. (a) PPAR*γ* and cleaved caspase-3 protein expression in PPAR*γ* regulation groups. (b) PDCD4 protein expression in PPAR*γ* regulation groups. (c) PDCD4 and cleaved caspase-3 protein expression in miR-21 regulation groups (a represents comparison with NC group, *p* < 0.05; b represents comparison with HR group, *p* < 0.05; c represents comparison with sh-PPAR*γ* group, *p* < 0.05; d represents comparison with OE-PPAR*γ* group, *p* < 0.05). Supplementary Figure 2: expression of PDCD4 and cleaved caspase-3 proteins (a represents comparison with NC group, *p* < 0.05; b represents comparison with HR group, *p* < 0.05; c represents comparison with mimic group, *p* < 0.05; d represents comparison with inhibitor group, *p* < 0.05).
