# Supplementary material for: Protective Effects of PPARγ on Renal Ischemia-Reperfusion Injury by Regulating miR-21
Source: Oxid Med Cell Longev. 2022 Aug 30;2022:7142314. doi: 10.1155/2022/7142314 (PMC9448582; doi:10.1155/2022/7142314)
Supplement: Supplementary Materials — Supplementary Figure 1: expression of PPARγ, PDCD4, and cleaved caspase-3 proteins. (a) PPARγ and cleaved caspase-3 protein expression in PPARγ regulation groups. (b) PDCD4 protein expression in PPARγ regulation groups. (c) PDCD4 and cleaved caspase-3 protein expression in miR-21 regulation groups (a represents comparison with NC group, p < 0.05; b represents comparison with HR group, p < 0.05; c represents comparison with sh-PPARγ group, p < 0.05; d represents comparison with OE-PPARγ group, p < 0.05). Supplementary Figure 2: expression of PDCD4 and cleaved caspase-3 proteins (a represents comparison with NC group, p < 0.05; b represents comparison with HR group, p < 0.05; c represents comparison with mimic group, p < 0.05; d represents comparison with inhibitor group, p < 0.05). [file 7142314.f1.docx]

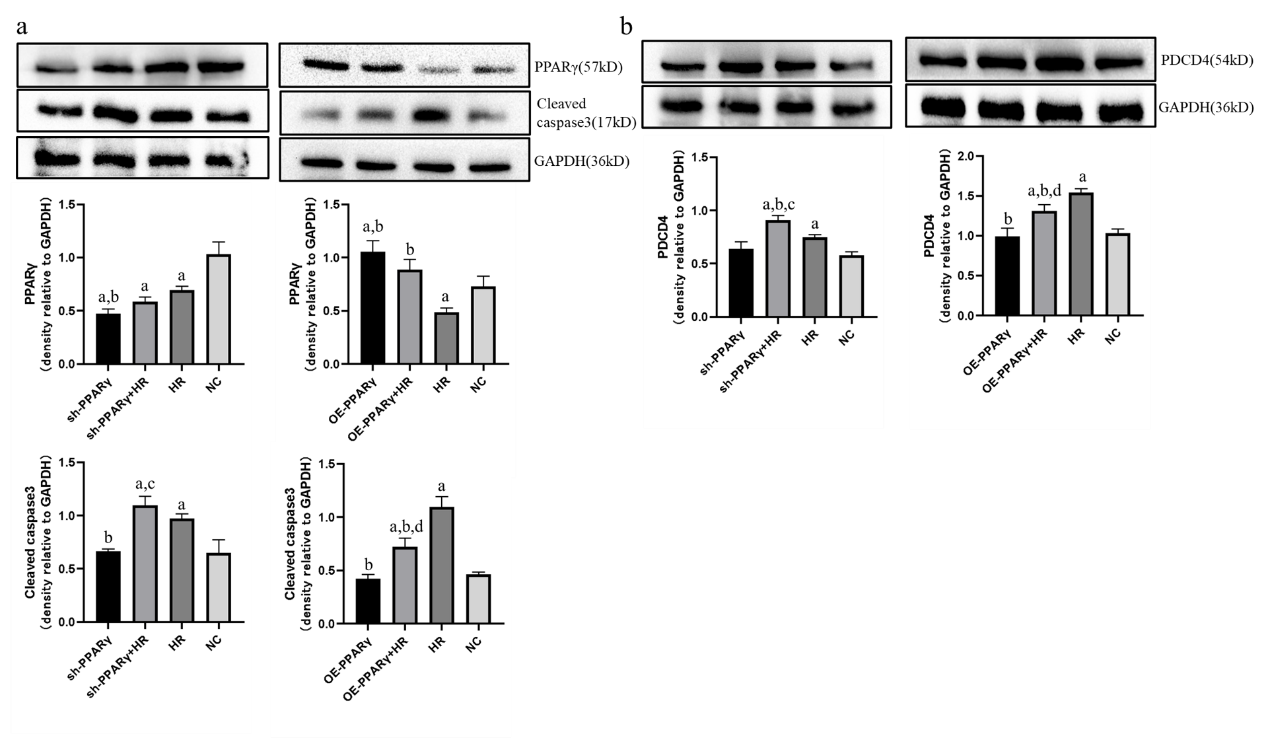
 Supplementary figure1. Expression of PPARγ, PDCD4 and cleaved caspase3 proteins. a PPARγ and cleaved caspase3 proteins expression in PPARγ regulation groups. b PDCD4 proteins expression in PPARγ regulation groups. c PDCD4 and cleaved caspase3 proteins expression in miR-21 regulation groups. (a represents comparison with NC group, p<0.05; b represents comparison with HR group, p<0.05; c represents comparison with sh-PPARγ group, p<0.05; d represents comparison with OE-PPARγ group, p<0.05.)


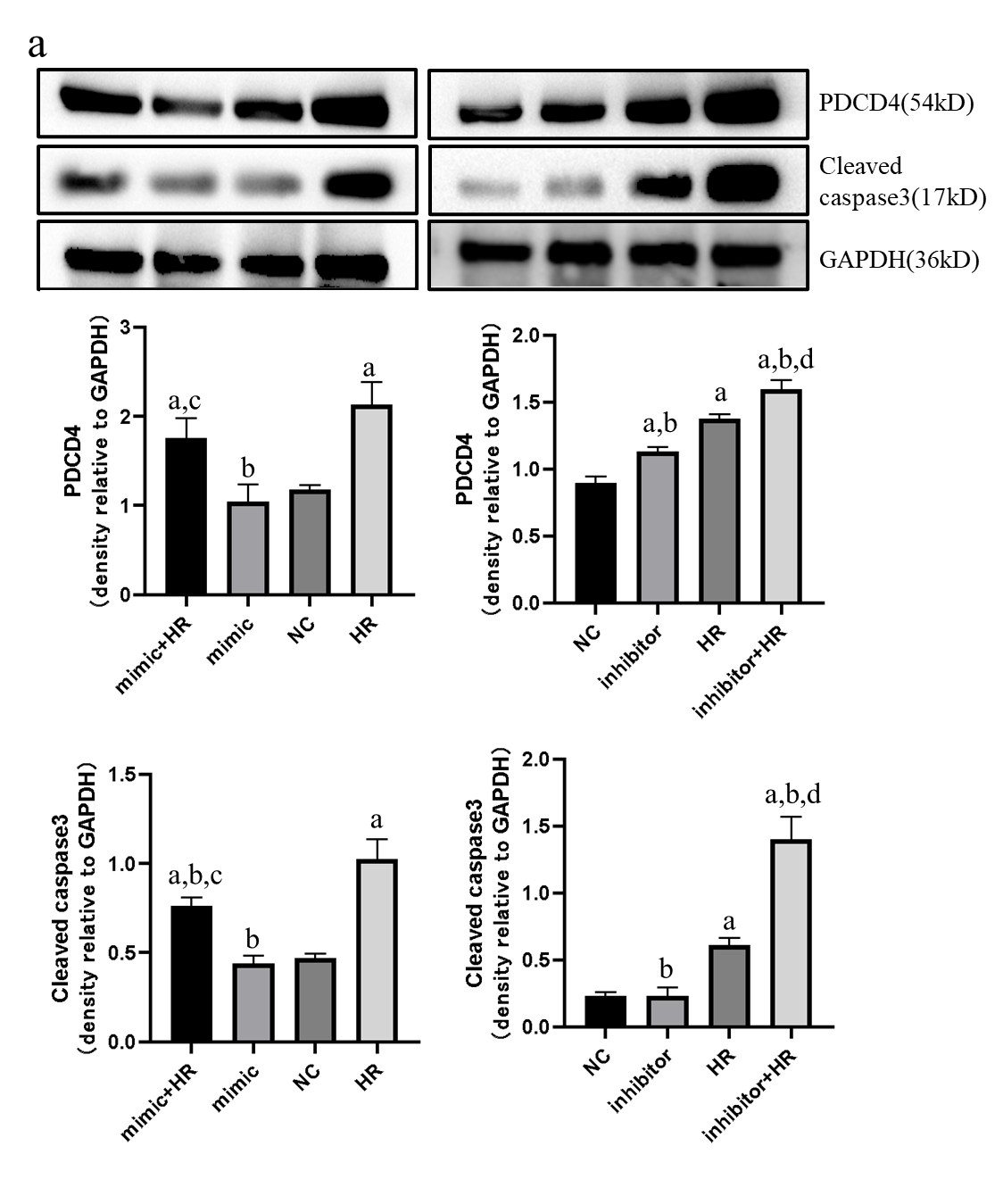


Supplementary figure2. Expression of PDCD4 and cleaved caspase3 proteins. (a represents comparison with NC group, p<0.05; b represents comparison with HR group, p<0.05; c represents comparison with mimic group, p<0.05; d represents comparison with inhibitor group, p<0.05.)
